# Activation of α7 nAChR by PNU-282987 improves synaptic and cognitive functions through restoring the expression of synaptic-associated proteins and the CaM-CaMKII-CREB signaling pathway

**DOI:** 10.18632/aging.102640

**Published:** 2020-01-06

**Authors:** Xiao-Ling Wang, Yu-Xin Deng, Yu-Mei Gao, Yang-Ting Dong, Fan Wang, Zhi-Zhong Guan, Wei Hong, Xiao-Lan Qi

**Affiliations:** 1 Key Laboratory of Endemic and Ethnic Diseases, Guizhou Medical University, Ministry of Education, Guiyang 550004, P.R. China; 2 Key Laboratory of Medical Molecular Biology, Guizhou Medical University, Guiyang 550004, P.R. China; 3 School of Basic Medical Sciences, Guizhou Medical University, Guiyang 550004, P.R. China; 4 Department of Neurosurgery, Affiliated Hospital of Guizhou Medical University, Guiyang 550004, P.R. China; 5 Department of Pathology, Affiliated Hospital of Guizhou Medical University, Guiyang 550004, P. R. China

**Keywords:** α7 nAChR, β-amyloid peptide, synapse, CaM-CaMKII-CREB signaling pathway, Alzheimer’s disease

## Abstract

Ligands of nicotinic acetylcholine receptors (nAChRs) are widely considered as potential therapeutic agents. The present study used primary hippocampus cells and APPswe/PSEN1dE9 double-transgenic mice models to study the possible therapeutic effect and underlying mechanism of the specific activation of α7 nAChR by PNU-282987 in the pathogenesis of Alzheimer’s disease. The results indicated that activation of α7 nAChR attenuated the Aβ-induced cell apoptosis, decreased the deposition of Aβ, increased the expression of synaptic-associated proteins, and maintained synaptic morphology. Furthermore, in the APP/PS1_DT mice model, activation of α7 nAChR attenuated Aβ-induced synaptic loss, reduced the deposition of Aβ in the hippocampus, maintained the integral structure of hippocampus-derived synapse, and activated the calmodulin (CaM)-calmodulin-dependent protein kinase II (CaMKII)-cAMP response element-binding protein signaling pathway by upregulation of its key signaling proteins. In addition, activation of α7 nAChR improved the learning and memory abilities of the APP/PS1_DT mice. Collectively, the activation of α7 nAChR by PNU-282987 attenuated the toxic effect of Aβ *in vivo* and *in vitro*, which including reduced deposition of Aβ in the hippocampus, maintained synaptic morphology by partially reversing the expression levels of synaptic-associated proteins, activation of the Ca^2+^ signaling pathway, and improvement of the cognitive abilities of APP/PS1_DT mice.

## INTRODUCTION

Alzheimer’s disease (AD) is the most common type of neurodegenerative disorder. The main clinical manifestations of AD patients are progressive memory decline, abnormal brain function and a decline in social adaptability. The pathological features of AD include the formation of extracellular senile plaques (SPs), intraneuronal neurofibrillary tangles aggregating of hyperphosphorylated tau protein and the loss of neurons [1]. SPs mainly consist of extracellular amyloid β (Aβ), which contains 38-43 amino acids and plays a key role in the pathogenesis of AD [2]. Deposition of Aβ in the human brain is considered to be associated with cross-sectional synaptic network dysfunction, progressive brain atrophy, an imbalance in neuronal calcium homeostasis and longitudinal cognitive decline [3]. Therefore, investigating methods to prevent the toxic effects of Aβ could be one of the promising strategies of treating AD patients.

Nowadays, multiple lines of transgenic mice have been developed to serve as animal models to study the progress of AD. APPswe/PSEN1dE9 double-transgenic mice (APP/PS_DT) harbor a mutated human amyloid precursor (APPswe, Swedish mutation) and human presenilin (PS DeltaE9) genes. A Swedish mutation of the amyloid precursor protein (APP) gene causes abnormal metabolism of APP and leads to excessive deposition of Aβ in the brain, which can induce neuronal apoptosis and necrosis [6]. Mutation of the PS1 gene can change the activity of γ-secretase and increase Aβ_42_ production [7, 8]. Double mutation of APP and PS1 genes in APP/PS1_DT mice leads to the accumulation of Aβ in the brain of these mice, thus causes deficits in learning and memory.

The neuropathological process of AD patients is highly associated with synaptic impairment. Synapses are the junction between two neurons and are the functionally connected parts of neurons, which serve a key role in the transmission of information. The primary function of the synapse is to mediate intercellular communication by releasing neurotransmitters from the presynaptic terminal and to modulate neural plasticity. The synaptic connection is an essential part of neurons that transmits information and is also the basis of learning and memory abilities. Numerous studies have also demonstrated that the decline of cognitive function in patients with AD is closely related to the impairment of synaptic function [15, 16]. Studies on the postmortem brains of individuals with AD, as well as on animal models connected with the disease, indicate that synapses are affected at the earliest stages of the neurodegenerative processes [4]. Loss of synapses in the brain tissues of patients with AD is associated with cognitive impairment [5].

Furthermore, nicotinic acetylcholine receptors (nAChRs) are involved in a variety of vital physiological processes, particularly in memory and cognition. Receptor-ligand binding studies have demonstrated that AD-associated loss of nAChRs is positively correlated with the presence of SPs in the temporal lobe [6]. nAChRs, including hetero- and homo-pentameric subtypes, consist of different combination of α (α2-α10) and β (β2-β4) subunits. In the human brain, α4β2 and α7 are located in cortical, striatal and hippocampal regions, which are involved in memory and cognition, and also the only subtypes of nAChRs that have been implicated in the pathogenesis of AD. A previous study reported that the APP-a7KO model (Tg2576 mice expressing the human amyloid precursor protein sequence with the Swedish mutation crossed with α7 nAChRs null-mutant) have worsened cognitive deficits when α7 nAChR is absent, and a decrease of α7 nAChRs levels are associated with synaptic damage in AD patients [19]. Furthermore, using specific agonists, which stabilize α7 nAChRs in the open state, has been shown to improve cognitive impairment in different AD models [7].

As activation of α7 nAChRs has a potentially beneficial effect on patients who suffer from cognitive impairments, extensive studies have been performed to study the underlying mechanism of the neural protective effects of α7 nAChRs agonist. It has been reported that nicotine, an unselective α7 nAChRs agonist, protects synapses from morphological destruction and synaptic impairment, which is exerted by Aβ oligomers. Activation of α7 nAChRs by agonist also prevents both early postsynaptic and late presynaptic impairments induced by Aβ via the α7 nAChRs/phosphatidylinositol-3 kinase (PI3K) signaling pathway and cross-talk with the Wnt/β-catenin pathway. Many selective agonists have also been used to activate α7 nAChRs as a potential treatment for patients with AD, including CCMI and PNU-1206596 [8]. Recently, a novel α7 nAChR selective agonist (PNU-282987) has been demonstrated to improve motor ataxia and anxiolytic effect in an AD mouse model [9]. It also has been reported that activation of α7 nAChRs by PNU-282987 can be used to ameliorate cognitive symptoms in schizophrenia and depression [7]. However, the underlying mechanism of PNU-282987 on synaptic changes and its neuroprotective signaling pathway are not fully understood.

In the present study, both primary hippocampus neuron cells and APP/PS1_DT mice models were used to investigate the effects of α7 nAChRs activation by PNU-282987 on synaptic morphology, the expression levels of synaptic-associated proteins, cognitive functions and the Ca^2+^ signaling pathway. The present results may provide a theoretical basis for further elucidating the pathogenesis of AD. Furthermore, it may shed light on the functions of α7 nAChRs during the development of AD.

## RESULTS

### Activation of α7 nAChR attenuates Aβ-induced apoptosis

**Figure 1:**
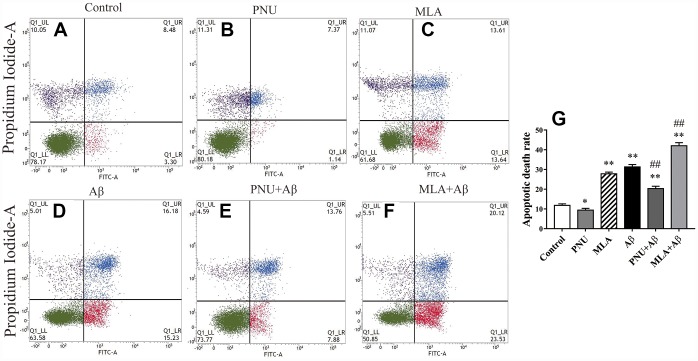
**Activation of α7 nAChR attenuates apoptosis induced by Aβ oligomers.** (**A**) The primary hippocampus cell group (Control), (**B**) the primary hippocampus cells treated with PNU (PNU), (**C**) the primary hippocampus cell treated with MLA (MLA), (**D**) the primary hippocampus cell treated with Aβ (Aβ), (**E**) the primary hippocampus treated with PNU and Aβ (PNU+Aβ), (**F**) the primary hippocampus treated with MLA and Aβ (MLA+Aβ) and (**G**) a histogram presenting the apoptotic death rates of the different groups. (**C** and **D**) The apoptosis rate of primary neuronal cells treated with MLA and Aβ oligomers was significantly increased, (**E**) while α7 nAChR attenuated apoptosis induced by Aβ oligomers. Data are presented as the mean ± standard deviation. ^*^P<0.05, ^**^P<0.01 vs. control group; ^#^P<0.05, ^##^P<0.01 vs. Aβ group.

To test whether Aβ oligomers increase the apoptosis rate of neuronal cells, flow cytometry was used to quantify the percentage of apoptotic cells by using the FITC Annexin V Apoptosis Detection Kit. The results demonstrated that the apoptosis rate of Aβ oligomer and MLA (an antagonist of α7 nAChR)-treated primary neuronal cells was significantly increased compared with the control group. Whereas PNU-282987 attenuated Aβ-induced apoptosis and MLA amplified Aβ-induced apoptosis (Figure 1).

### Activation of α7 nAChR enhances learning and memory abilities in APP/PS1_DT mice

**Figure 2:**
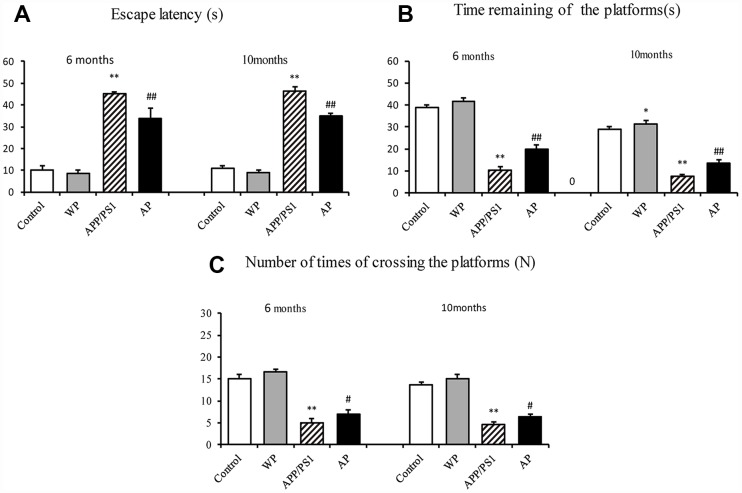
**Activation of α7 nAChR alleviates cognitive deficits in APP/PS1_DT mice.** (**A**) Escape latency of mice from different groups. (**B**) Times of staying in the platform (**C**) The numbers of crossing the platform. The abscissa indicate the wild-type C57 mice injected with saline (control), wild-type C57 mice injected with PNU-282987 (WP), APP/PS1_DT mice injected with saline (APP/PS1) and APP/PS1_DT mice injected with PNU-282987 (AP). The Morris water maze demonstrated that both spatial learning, and spatial memory abilities were declined in 6- and 10-months-old APP/PS1_DT mice, while α7 nAChR alleviated cognitive deficits in APP/PS1_DT mice. These results indicate that the expression level of α7 nAChR is associated with improved cognitive function in the APP/PS1_DT mice. Data are presented as the mean ± standard deviation. ^*^P<0.05, ^**^P<0.01 vs. control group; ^#^P<0.05, ^##^P<0.01 vs. APP/PS1 group. s, seconds; N, times.

To examine whether activation of α7 nAChR by PNU-282987 alleviates the cognitive deficits in APP/PS1_DT mice, the present study measured the potential of α7 nAChR to alleviate AD-induced declines in spatial memory and learning ability by using the Morris water maze [10]. The APP/PS1 group demonstrated a longer escape latency compared with the control group. However, the increased escape latency of APP/PS1_DT mice was alleviated by the administration of PNU-282987 (AP group) (Figure 2A). The platform in the water maze was removed from the pool on day 5 of testing, and all groups of mice were allowed to swim for one minute. The time of staying at the platform and the number crossing the platform of PNU-282987-treated mice (AP group) was dramatically increased compared with the APP/PS1_DT mice (APP/PS1 group; Figure 2B and 2C). These results suggested that activation of α7 nAChR improves the spatial learning and memory disorders in APP/PS1_DT mice.

### Activation of α7 nAChR reduces the deposition of Aβ in the hippocampus of APP/PS1_DT mice

**Figure 3:**
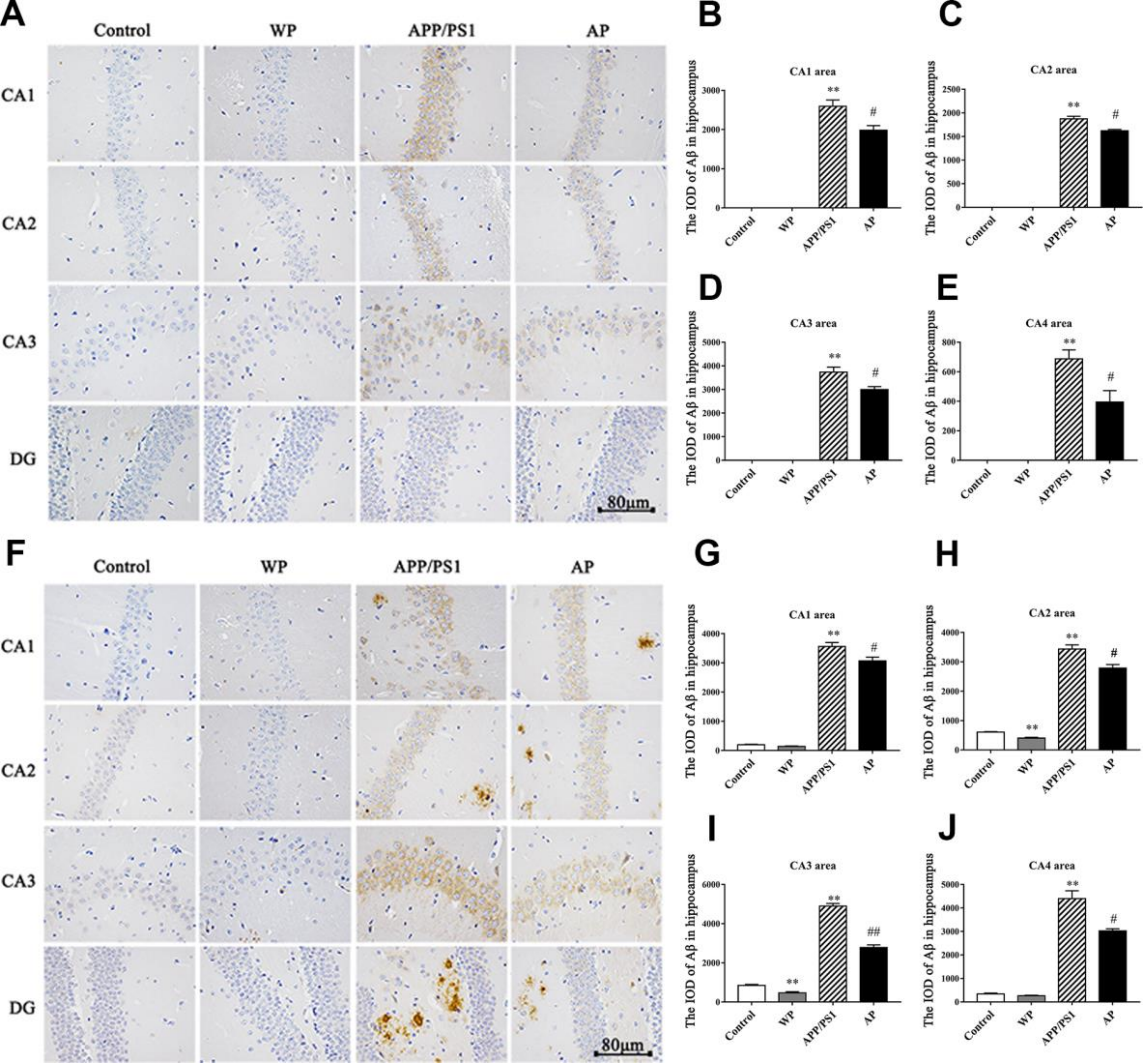
**Activation of α7 nAChR reduces the deposition of Aβ in the hippocampus of the APP/PS1_DT mice.** The deposition of Aβ in the hippocampus of APP/PS1_DT mice (**A**) 6 months old and (**F**) 10 months old. The IOD of Aβ in the hippocampus area of 6 months old mice in the (**B**) CA1 area, (**C**) CA2 area, (**D**) CA3 area, (**E**) CA4 area, and 10 months old mice in the (**G**) CA1 area, (**H**) CA2 area, (**I**) CA3 area and (**J**) CA4 area. Control, WT C57 mice injected with saline; WP, WT mice injected with PNU; APP/PS1, APP/PS1_DT mice injected with saline; AP, APP/PS1_DT mice injected with PNU. CA1, CA2, CA3 and DG indicate the CA1 area, CA2 area, CA3 area and DG area of the hippocampus, respectively. Compared with the control group, Aβ deposition in the hippocampus of APP/PS1_DT mice was increased significantly, and this trend was partially reversed by PNU treatment (AP group). The results demonstrated that α7 nAChR partially reduced the deposition of Aβ in the hippocampus of the APP/PS1_DT mice. Data are presented as the mean ± standard deviation. ^*^P<0.05, ^**^P<0.01 vs. control group; ^#^P<0.05, ^##^P<0.01 vs. APP/PS1 group.

Aβ plays a vital role in the cognitive dysfunction of AD. Thus, the present study observed the expression of Aβ by immunohistochemical staining. As presented in Figure 3 (Figure 3A and 3F for 6- and 10-month old mice, respectively), the levels of Aβ integrated optical density (IOD) were significantly increased in the APP/PS1 group, both in the 6-month-old and 10-month-old APP/PS1_DT mice groups (Figure 3A and 3F). Whereas Aβ deposition was partially alleviated by PNU-282987 treatment (Figure 3B–3E and 3G–3J). The results indicated that α7 nAChR could reduce the deposition of Aβ in the hippocampus of APP/PS1_DT mice, thus, alleviate the toxic effect of Aβ on neuronal cells.

### Activation of α7 nAChR promotes the expression of synaptic-associated proteins in Aβ oligomers treated neurons

**Figure 4:**
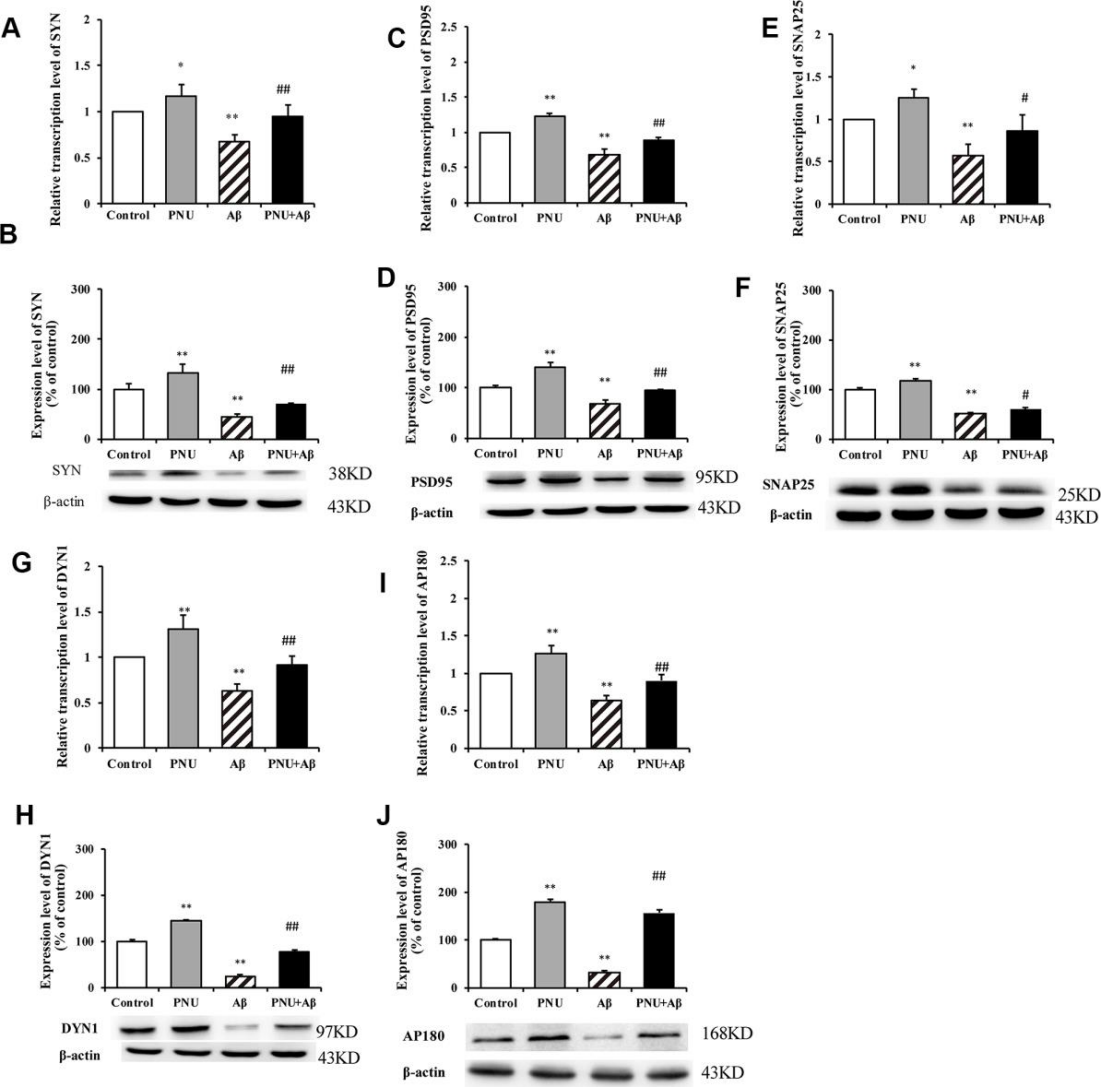
**Activation of α7 nAChR promotes the expression of synaptic-associated proteins in Aβ oligomer-treated neurons.** The x-axis labels are the neurons isolated from the WT rat (control), the WT neuron cells treated with PNU (PNU), the WT neuron cells treated with Aβ (Aβ) and the WT neuron cells treated with PNU and Aβ (PNU+Aβ). The y-axis indicates the relative level of mRNA or protein (% of control). Detection of SYN (**A**) mRNA and (**B**) protein; PSD95 (**C**) mRNA and (**D**) protein; SNAP25 (**E**) mRNA and (**F**) protein; DYN1 (**G**) mRNA and (**H**) protein; AP180 (**I**) mRNA and (**J**) protein. The relative level in each group was measured by RT-qPCR and western blot analysis, and β-actin was used as an internal control. The results demonstrated that the protein expression levels of SYN, PSD95, SNAP25, DYN1 and AP180 were significantly decreased in Aβ oligomer-treated neurons, and this decrease was partially reversed by PNU treatment. Data are presented as the mean ± standard deviation. ^*^P<0.05, ^**^P<0.01 vs. control group; ^#^P<0.05, ^##^P<0.01 vs. Aβ.

To explore whether α7 nAChR could restore synaptic-associated proteins in Aβ oligomers-treated neurons, reverse transcription-quantitative PCR (RT-qPCR) and western blot analysis were used to quantify the expression of synaptophysin (SYN), postsynaptic density of 95 (PSD95), synaptosomal-associated protein of 25 kD (SNAP25), dynamin 1 (DYN1) and adaptor protein of 180 (AP180) in hippocampal neurons. As presented in Figure 4, compared with the control group, the relative transcription levels of SYN (Figure 4A), PSD95 (Figure 4C), SNAP25 (Figure 4E), DYN1 (Figure 4G), AP180 (Figure 4I) were significantly decreased in Aβ oligomer-treated neurons. Correspondingly, compared with the control group, the protein levels of SYN (Figure 4B), PSD95 (Figure 4D), SNAP25 (Figure 4F), DYN1 (Figure 4H), AP180 (Figure 4J) were also significantly decreased in Aβ oligomer-treated neurons. Whereas, the decrease of these protein levels was partially reversed by PNU-282987 treatment. To verify that the neuroprotective effect is α7 nAChRs-dependent, the α7 nAChR-selective competitive antagonist methyllycaconitine (MLA) was used to reduce activation of α7 nAChRs. As presented in Supplementary Figure 4, the protein expression levels of SYN (Supplementary Figure 4A), PSD95 (Supplementary Figure 4B) and DYN1 (Supplementary Figure 4D) in the MLA+Aβ group (WT neuron cells treated with 100 nmol/l MLA and 0.5 μmol/l Aβ for 24 hours) were decreased compared with the Aβ group (WT neuron cells treated with 0.5 μmol/l Aβ for 24 hours). Notably, there were no significant differences in the protein levels of SNAP25 (Supplementary Figure 4C) and AP180 (Supplementary Figure 4E) compared with the Aβ group. These results indicate alleviation of synaptic dysfunction *in vitro* by PNU-282987 is α7 nAChR-dependent.

### Activation of α7 nAChR increases the expression of synaptic-associated proteins in the hippocampus of APP/PS1_DT mice

**Figure 5:**
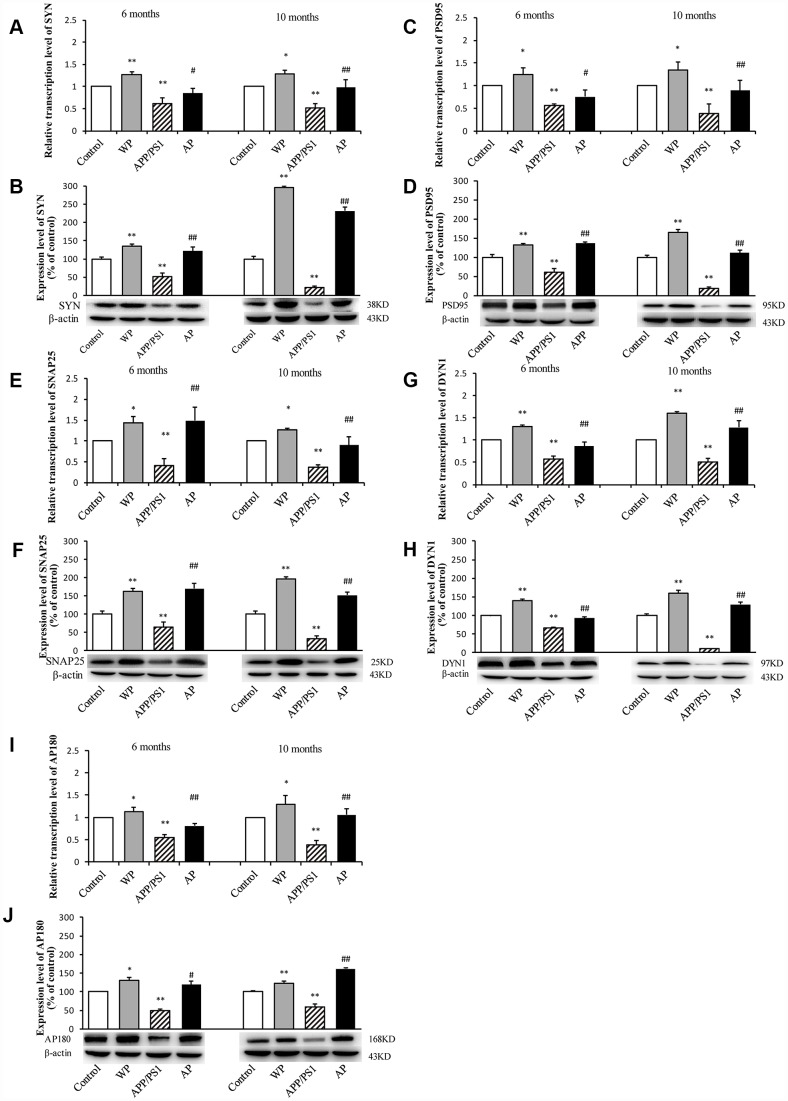
**Activation of α7 nAChR increases the expression of synaptic-associated proteins in the hippocampus of APP/PS1_DT mice**. The x-axes are the WT mice (control), the WT mice treated with PNU (WP), the APP/PS1_DT mice (APP/PS1) and the APP/PS1_DT mice treated with PNU (AP). The y-axes are the relative level of mRNA or protein (% of control group). Detection of SYN (**A**) mRNA and (**B**) protein; PSD95 (**C**) mRNA and (**D**) protein; SNAP25 (**E**) mRNA and (**F**) protein; DYN1 (**G**) mRNA and (**H**) protein; AP180 (**I**) mRNA and (**J**) protein by RT-qPCR and western blot analysis. Protein expression levels were detected by western blot analysis (β-actin was used as an internal control). RT-qPCR and western blot analysis demonstrated that the expression levels of SYN, PSD95, SNAP25, DYN1 and AP180 in the hippocampus of APP/PS1_DT mice were significantly decreased compared with the control group, and this decreasing trend was partially reversed by PNU treatment. Data are presented as the mean ± standard deviation. ^*^P<0.05, ^**^P<0.01 vs. control group; ^#^P<0.05, ^##^P<0.01 vs. APP/PS1 group.

The present study subsequently evaluated the expression of synaptic-associated proteins (SYN, PSD95, SNAP25, DYN1 and AP180) at the mRNA and protein level in the hippocampus of APP/PS1_DT mice (6- and 10-months old). As shown in Figure 5, RT-qPCR and western blot analysis revealed that in APP/PS1_DT group, the SYN, PSD95, SNAP25, DYN1 and AP180 mRNA (Figure 5A, 5C, 5E, 5G and 5I) and protein levels (Figure 5B, 5D, 5F, 5H and 5J) in the hippocampus were significantly reduced. Whereas, following PNU-282987 treatment, the expression levels of SYN, PSD95, SNAP25, DYN1 and AP180 were significantly increased compared with the APP/PS1_DT group. These data indicated that α7 nAChR partially reverses the loss of synaptic-associated proteins.

### Expression of SYN in primary hippocampus neurons detected by immunofluorescence

**Figure 6:**
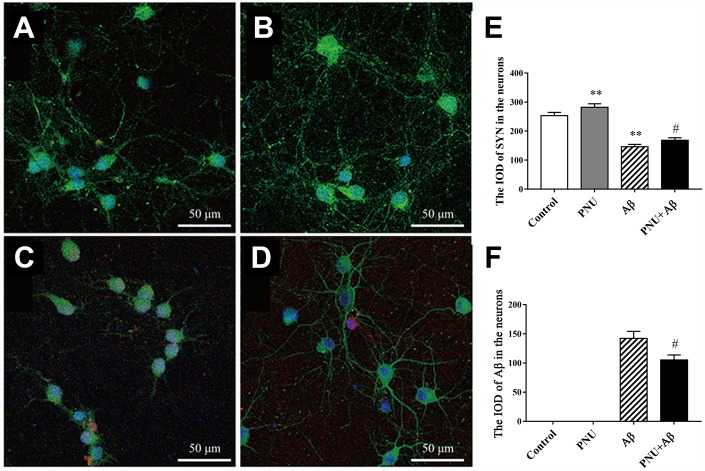
**Activation of α7 nAChR promotes the expression of SYN in Aβ oligomer-treated neurons.** (**A**) Hippocampus cells isolated from WT mice (control), (**B**) control treated with PNU (PNU), (**C**) control treated with Aβ (Aβ) and (**D**) control treated with PNU and Aβ (PNU+Aβ). (**E**) The IOD of SYN in the hippocampus neurons. (**F**) The IOD of Aβ in the hippocampus neurons. SYN, Aβ oligomers and the nuclei were labeled green, red and blue, respectively. Magnification, x200. Compared with the (**A**) control group, the expression level of SYN (green) was significantly decreased in the (**C**) Aβ oligomer group, (**D**) and this decreasing was partially reversed by PNU treatment. Data are presented as the mean ± standard deviation. ^*^P<0.05, ^**^P<0.01 vs. control group; ^#^P<0.05, ^##^P<0.01 vs. Aβ group.

Evidence has shown that the levels of PSD95 and SYN are reduced in AD transgenic mice models [11, 12] and the brains of patients with AD [13]. SYN and PSD95 are markers of the pre- and post-synapse, respectively. Furthermore, both *in vivo* and *in vitro* experiments have shown that Aβ monomer can lead to synaptic plasticity damage and synaptic loss. The Aβ oligomers can cause synaptic dysfunction [14]. The present study used immunofluorescence to investigate whether α7 nAChR could restore SYN expression in Aβ oligomers-treated neurons. As shown in Figure 6, the expression level of SYN was significantly decreased in the Aβ oligomer-treated group, and this decreasing trend was partially reversed by PNU-282987 treatment (Figure 6E and 6F). This result indicates that α7 nAChR could attenuate synaptic loss *in vitro*.

### Expression of SYN in a mice model detected by immunofluorescence staining

**Figure 7:**
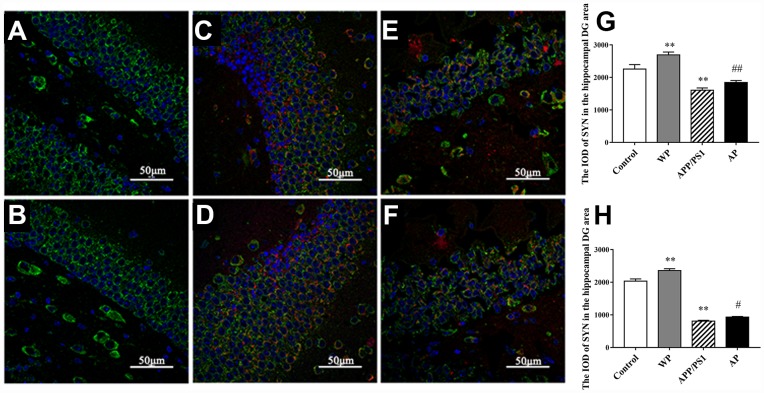
**Activation of α7 nAChR attenuates the expression of SYN in the hippocampus of APP/PS1_DT mice.** (**A**) Hippocampus cells isolated from WT mice (Control); (**B**) Controls treated with PNU (WP); (**C**) 6-month-old APP/PS1_DT mice; (**D**) 6-month-old APP/PS1_DT mice treated with PNU; (**E**) 10-month-old APP /PS1_DT mice and (**F**) 10-month-old APP/PS1 mice treated with PNU. (**G**) The IOD of SYN in the hippocampus DG area, which was collected from 6-month-old mice. (**H**) The IOD of SYN in the hippocampus DG area, which was collected from 10-month-old mice. SYN, Aβ and nucleus are labeled in green, red and blue, respectively. Magnification, x200. Compared with the (**A**) control group, the expression level of SYN was significantly decreased in the hippocampus DG area of (**C**) 6- and (**E**) 10- month-old APP/PS1_DT mice. (**D** and **F**) The Aβ distribution and SYN expression were negatively correlated, and the decrease of SYN could be partially reversed by PNU treatment. Data are presented as the mean ± standard deviation. ^*^P<0.05, ^**^P<0.01 vs. control group; ^#^P<0.05, ^##^P<0.01 vs. APP/PS1 group.

To investigate whether α7 nAChR could restore SYN expression in APP/PS1_DT mice, immunofluorescence was used to determine the expression of SYN in the hippocampus of the DG area. As presented in Figure 7, the expression level of SYN was significantly decreased in the hippocampus DG area of APP/PS1_DT mice (in both 6- and 10- months-old mice). The Aβ distribution and SYN expression were negatively correlated, and the reduction of SYN could be partially reversed by PNU-282987 treatment (Figure 7G and 7H). These results suggested that α7 nAChR partially attenuates synaptic loss in the hippocampus of APP/PS1_DT mice.

### Expression of PSD95 in the mice model detected by immunohistochemistry

**Figure 8:**
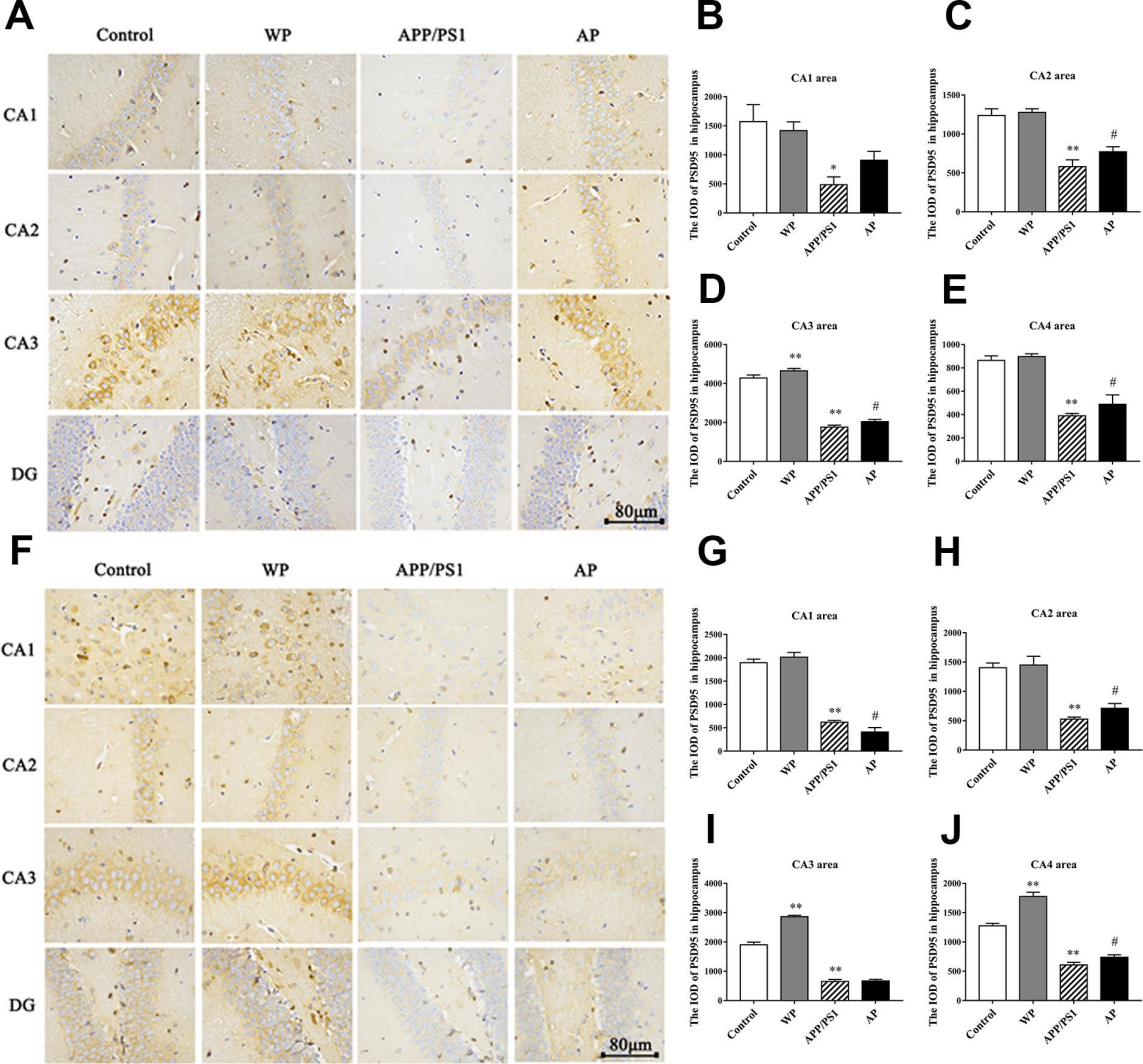
**Activation of α7 nAChRs increases the expression of PSD95 protein in the hippocampus of APP/PS1_DT mice.** The expression of PSD95 protein in the hippocampus of the APP/PS1_DT mice at (**A**) 6-months and (**F**) 10-months old. The IOD of PSD95 in hippocampus (**B**) CA1 area, (**C**) CA2 area, (**D**) CA3 area and (**E**) CA4 area of 6-month-old APP/PS1_DT mice. The IOD of PSD95 in hippocampus (**G**) CA1 area, (**H**) CA2 area, (**I**) CA3 area and (**J**) CA4 area of 10-month-old APP/PS1_DT mice. Control, WT C57 mice injected with saline; WP, WT mice injected with PNU; APP/PS1, APP/PS1_DT mice injected with saline; and AP, APP/PS1_DT mice injected with PNU. Compared with the control group, the expression of PSD95 protein in the hippocampus of the APP/PS1_DT mice group was significantly decreased and was partially reversed by PNU. This result suggested that activation of α7 nAChR partially reversed the expression level of PSD95 protein in the hippocampus of APP/PS1_DT mice. Data are presented as the mean ± standard deviation. ^*^P<0.05, ^**^P<0.01 vs. control group; ^#^P<0.05, ^##^P<0.01 vs. APP/PS1 group.

The PSD95 is closely related to synaptic fitness, and a decrease in its expression indicates synaptic malfunction [15]. The PSD95 interacts with a variety of post-synaptic membrane proteins, participates in the formation of synaptic connections and participates in post-synaptic signal transduction. Immunohistochemical staining was used to measure the expression of PSD95. The expression levels of PSD95 were significantly decreased in the 6-month-old APP/PS1_DT group (Figure 8A–8E), and the expression level of PSD95 was significantly reduced in the hippocampus of 10-month-old APP/PS1_DT mice (Figure 8F–8J). As expected, the expression level of PSD95 was partially reversed by PNU-282987 treatment (Figure 8B–8E and 8G–8J).

### Synapse morphology observed by transmission electron microscopy (TEM)

**Figure 9:**
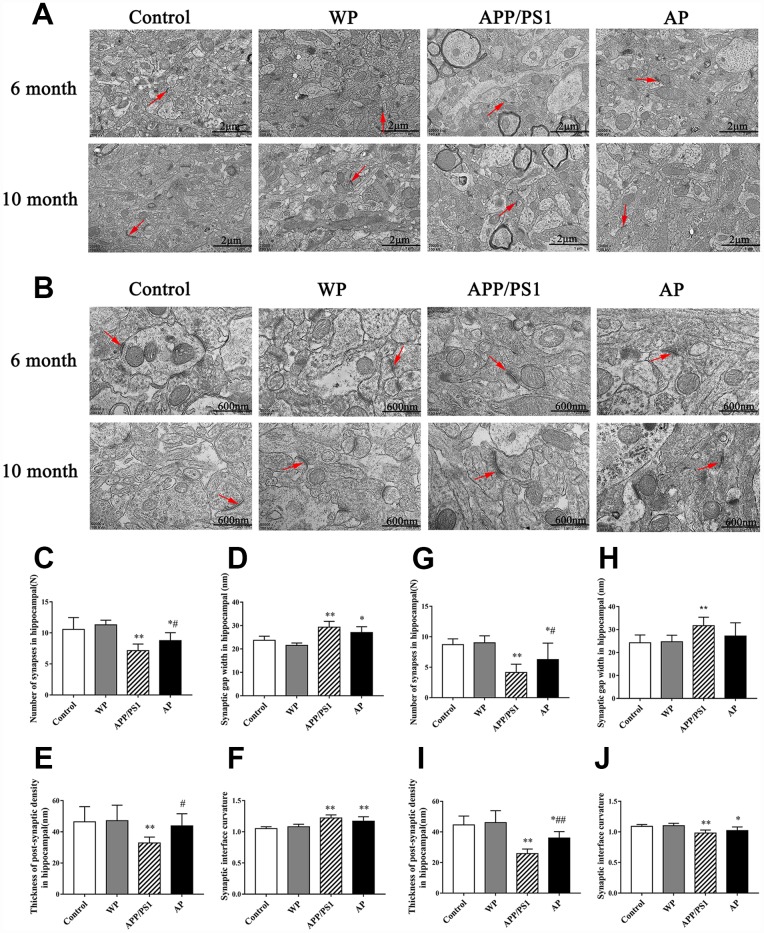
**Activation of α7 nAChR increases the number of synapses and maintains the structural integrity of synapses in the hippocampus of APP/PS1_DT mice.** The synapses were visualized using transmission electron microscopy. Control, the WT mice; WP, the WT treated with PNU; APP/PS1, the APP/PS1_DT mice; and AP, the APP/PS1_DT mice treated with PNU. Magnification, (**A**) x20 and (**B**) x50. The number and characteristics of synapses in (**C**–**F**) 6- and (**G**–**J**) 10-months-old mice are presented in the figure. Arrows indicate synapses. The results demonstrated that synapses underwent the following changes: (**C** and **G**) The number of synapses in both 6- and 10-month-old mice was decreased; (**D** and **H**) the synaptic gap became wider; (**E** and **I**) the post-synaptic dense substance in the hippocampus of the APP/PS1 group became thinner (vs. the control group) and the post-synaptic dense substance in the AP groups (either 6- or 10-month-old) were thicker than the APP/PS1 group; and (**F** and **J**) the curvature of the synaptic interface became smaller in 10-month-old mice. (**D**, **H**, **F** and **J**) Moreover, there were no significant differences in synaptic gap width and synaptic interface curvature between the APP/PS1 and AP groups. These results indicate that α7 nAChR is beneficial to maintain the integrity of the synaptic structure and increase the number of synapses, thus could improve synaptic functions. Data are presented as the mean ± standard deviation. ^*^P<0.05, ^**^P<0.01 vs. control group; ^#^P<0.05, ^##^P<0.01 vs. APP/PS1 group.

Synapses are the basic structure for the connection between neurons. In the central nervous system, the number of synapses directly affects the efficiency of information transmission. Synaptic transmission efficiency is correlated with synaptic gap width. Additionally, postsynaptic density and curvature regulates synaptic plasticity [16]. The image of TEM revealed that compared with the control group, the number of synapses and synaptic vesicles was significantly decreased, and activation of α7 nAChR increased the number of synapses in the hippocampus of APP/PS1_DT mice (Figure 9).

The hippocampal neurons of the 6-month and 10-month-old APP/PS1_DT mice underwent the following morphological changes: i) The number of synapses in both 6- and 10-months-old mice was decreased (Figure 9C and 9G); ii) synaptic gap became wider (Figure 9D and 9H); iii) the post-synaptic dense substance in the hippocampus of APP/PS1 group became thinner (vs. the control group) and the post-synaptic dense substance in the AP groups (either 6-month-old or 10-month-old) was thicker than in the APP/PS1 group (Figure 9E and 9I); and iv) the curvature of the synaptic interface became smaller in 10-month-old mice (Figure 9F and 9J). Furthermore, there were no significant differences in synaptic gap width and synaptic interface curvature between the APP/PS1 and AP groups (Figure 9D, 9F, 9H and 9J). These results indicated that α7 nAChR is beneficial to maintain the integrity of the synaptic structure and increase the number of synapses, thus could improve synaptic functions.

### Activation of α7 nAChR activated the CaM-CaMKII-CREB signaling pathway in Aβ oligomer-treated neurons

**Figure 10:**
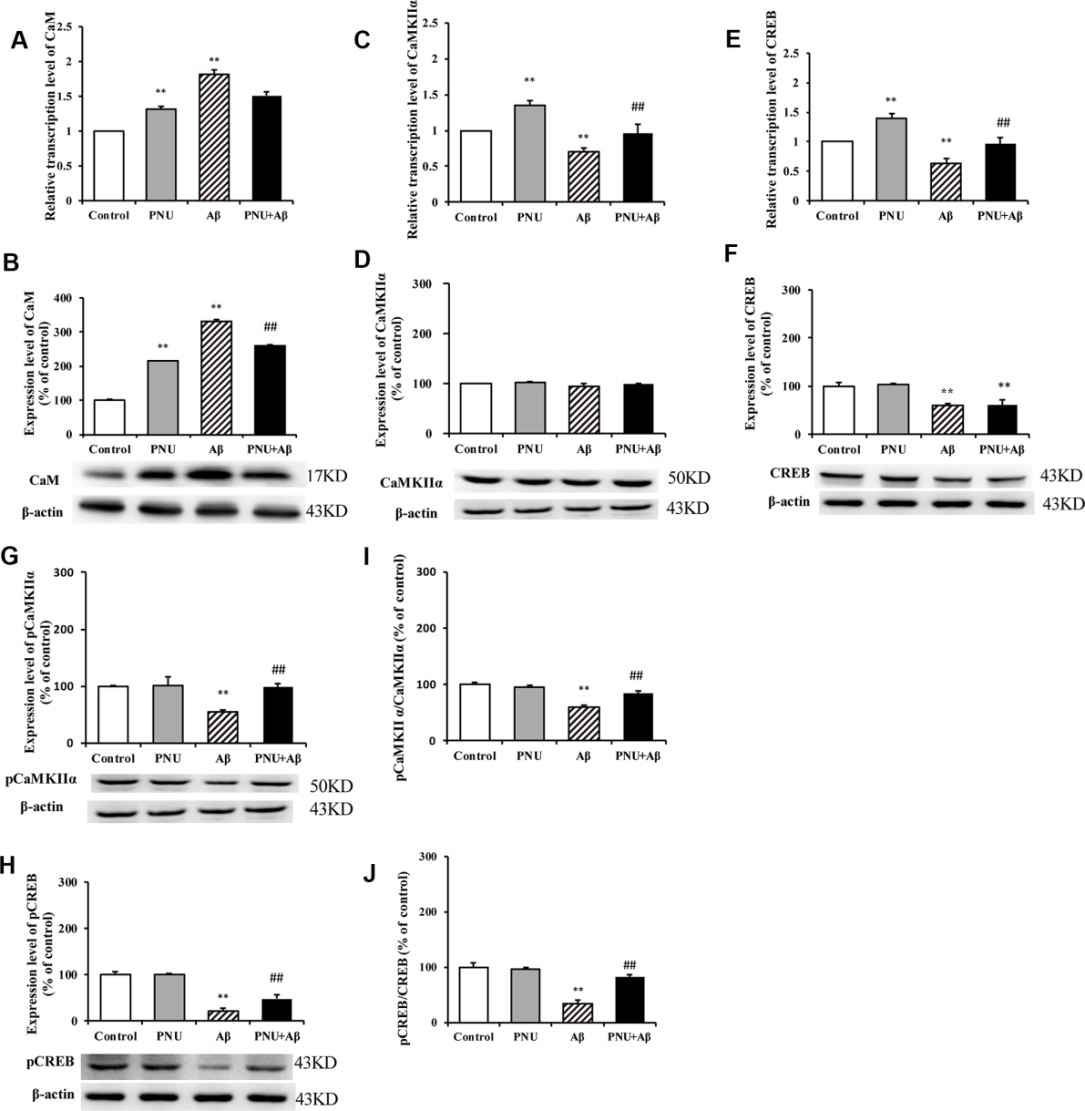
**Activation of α7 nAChR activates the CaM-CaMKII-CREB signaling pathway in Aβ oligomer-treated neurons.** The x-axis labels are Control, hippocampus cells from the WT rat; PNU, WT hippocampus cells treated with PNU; Aβ, the WT hippocampus cells treated with Aβ; and PNU+Aβ, the WT hippocampus cells treated with PNU and Aβ. The y-axis indicates the relative mRNA or protein levels as a percentage of the control. mRNA and protein expression levels in each group were measured by RT-qPCR and western blot analysis, respectively. Detection of the levels of CaM (**A**) mRNA and (**B**) protein, CaMKIIα (**C**) mRNA and (**D**) protein, CREB (**E**) mRNA and (**F**) protein, (**G**) p-CaMKIIα protein and (**H**) p-CREB protein, and the (**I**) p-CaMKIIα/CaMKIIα and (**J**) p-CREB/CREB ratios. The results demonstrated that the transcription of CaM and the protein level of CaM were significantly increased in the Aβ group, while the expression level of α7 nAChR was decreased. The transcription level of CaMKIIα was significantly decreased, and the expression levels of p-CaMKIIα, CREB and p-CREB, and p-CaMKIIα/CaMKIIα and p-CREB/CREB ratios were significantly decreased in the Aβ group. All these protein levels were largely restored following activation of α7 nAChR by PNU treatment. Data are presented as the mean ± standard deviation. ^*^P<0.05, ^**^P<0.01 vs. Control group; ^#^P<0.05, ^##^P<0.01 vs. Aβ.

The calcium disorder theory suggests that the concentration of calcium ions determines whether neuronal cells can produce excitatory and complete excitatory signals. The accumulation of Aβ in AD patients causes intracellular calcium (Ca^2+^) ion disorder; thus, damages synaptic plasticity [17]. Ca^2+^ can activate calpain kinase, which damages the cellular structure and leads to decreased cognitive ability in patients [18]. Therefore, the CaM-CaMKII-CREB signaling pathway, which regulates Ca^2+^ concentration in neuronal cells, plays an important role in synaptic degeneration and memory loss in AD, and it is also a potential therapeutic target for treating AD [19]. The present study investigated whether PNU-282987 activated the CaM- CaMKII-CREB signaling pathway in hippocampal neurons. As shown in Figure 10, the relative mRNA and protein levels of CaM (Figure 10A and 10B) were increased; however, the mRNA expression levels of CaMKIIα (Figure 10C) and CREB (Figure 10E) were decreased in the Aβ oligomer-treated neurons. The protein level of CaMKIIα (Figure 10D) remained unchanged, while the protein levels of CREB (Figure 10F), phosphorylated (p)-CaMKIIα (Figure 10G), p- CREB (Figure 10H), and the ratio of phosphorylated to non- phosphorylated proteins, p-CaMKIIα/ CaMKIIα (Figure 10I) and p-CREB/CREB (Figure 10J) were decreased in the Aβ oligomer-treated neurons group. Furthermore, the abundance of all proteins (except CaMKIIα) was largely restored following PNU-282987 treatment and the protein level of CaM (Figure 10B) was decreased.

### Activation of α7 nAChR activates the CaM-CaMKII-CREB signaling pathway in the hippocampus of APP/PS1_DT mice

**Figure 11:**
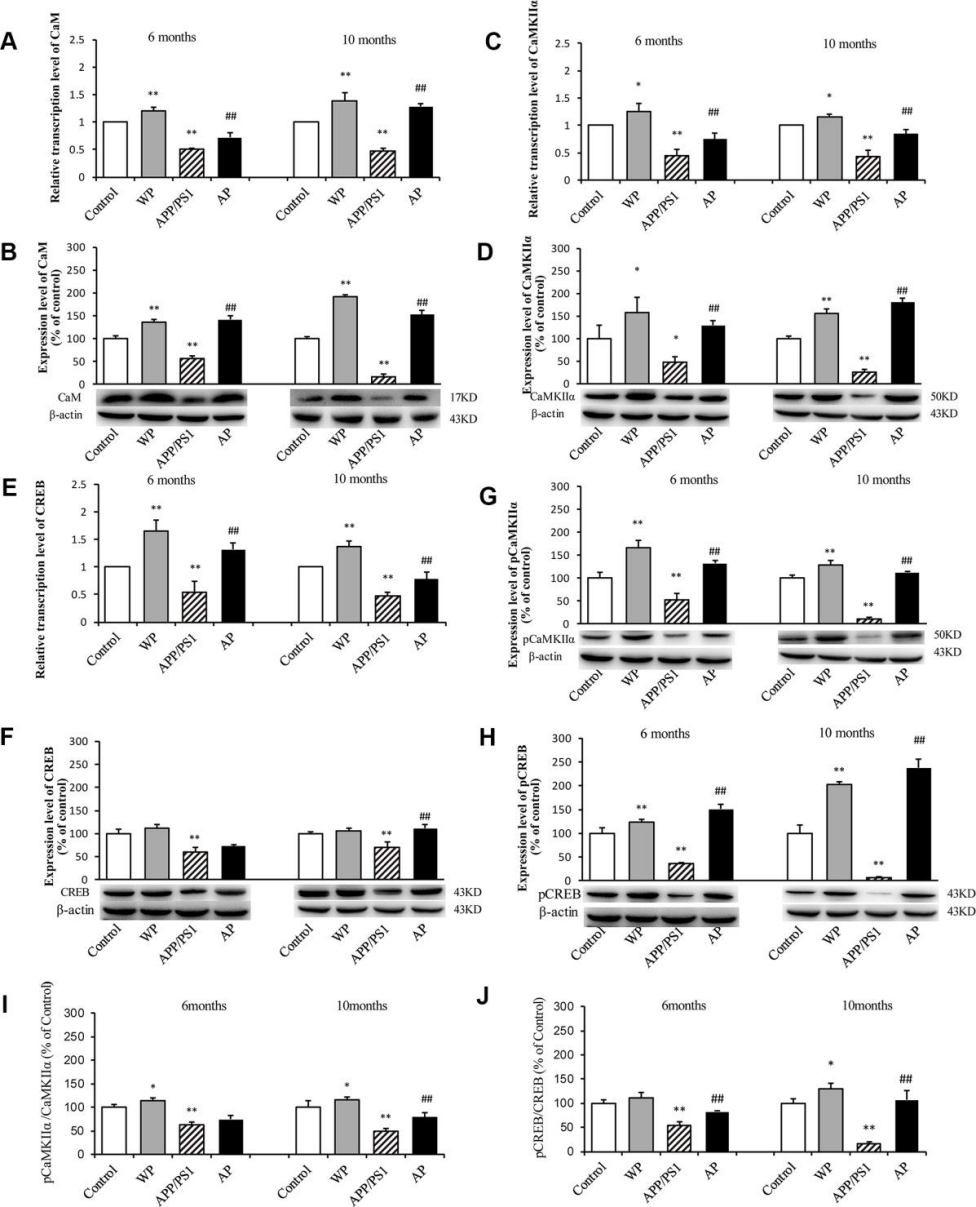
**Activation of α7 nAChR activates the CaM-CaMKII-CREB signaling pathway in the hippocampus of APP/PS1_DT mice.** The x-axis indicates the WT mice (control); WT mice treated with PNU (WP); APP/PS1_DT mice (APP/PS1); and APP/PS1_DT mice treated with PNU (AP). The y-axes indicate the relative mRNA and protein levels as a percentage of the control. Compared with the control group, the transcription levels of CaM (**A**), CaMKIIα (**C**) and CREB (**E**) were significantly decreased in the hippocampus of APP/PS1_DT mice. The protein expression levels of CaM (**B**), CaMKIIα (**D**), CREB (**F**), p-CaMKIIα (**G**) and p-CREB (**H**) were also significantly reduced. Moreover, the p-CaMKIIα/CaMKIIα (**I**) and p-CREB/CREB (**J**) ratios were significantly reduced. The protein expression levels of CaM, CaMKII and CREB in the hippocampus of APP/PS1_DT mice were significantly increased after PNU treatment (except CREB and CaMKIIα/CaMKIIα in 6-month mice in the AP group). Data are presented as the mean ± standard deviation. ^*^P<0.05, ^**^P<0.01 vs. control group; ^#^P<0.05, ^##^P<0.01 vs. APP/PS1 group.

The present study further investigated whether α7 nAChR can activate the CaM-CaMKII-CREB signaling pathway in APP/PS1_DT mice. As shown in Figure 11, compared with the control group, the transcription levels of CaM (Figure 11A), CaMKIIα (Figure 11C) and CREB (Figure 11E) were significantly decreased in the hippocampus of APP/PS1_DT mice. The protein expression levels of CaM (Figure 11B), CaMKIIα (Figure 11D), CREB (Figure 11F), p-CaMKIIα (Figure 11G) and p-CREB (Figure 11H) were also significantly reduced. Moreover, the p-CaMKIIα/CaMKIIα (Figure 11I) and p-CREB/CREB (Figure 11J) ratios were significantly reduced. The expression levels of CaM, CaMKII and CREB proteins in the hippocampus of APP/PS1_DT mice were significantly increased after PNU treatment (except CREB and CaMKIIα/CaMKIIα in 6-month mice in the AP group). These results suggested that α7 nAChR activates the CaM-CaMKII-CREB signaling pathway.

### Activation of α7 nAChR activates the calcium signaling pathway in KN93 treated neurons

**Figure 12:**
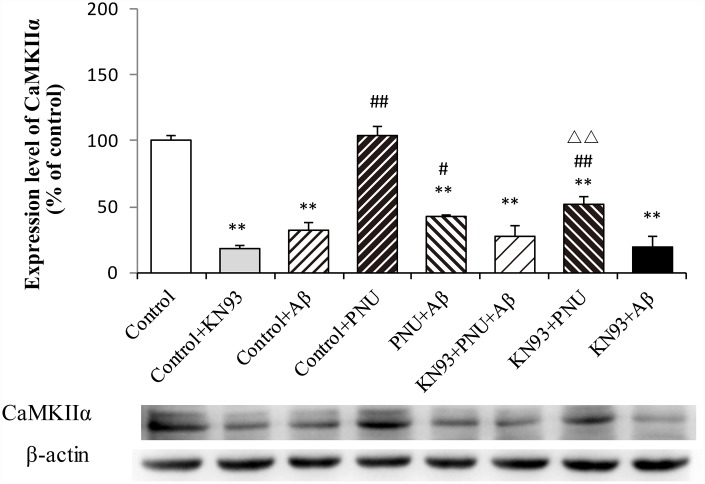
**Activation of α7 nAChR activates the calcium signaling pathway in Aβ oligomer and KN93-treated neurons.** Protein levels in each group were determined by western blot analysis and β-actin was used as an internal control. The x-axis indicates the Control, hippocampus cells of the WT rat; Control + KN93, the WT hippocampus cells treated with CaMKII inhibitor (KN93); Control + Aβ, the WT hippocampus cells treated with Aβ; Control + PNU, the WT hippocampus cells treated with PNU; PNU + Aβ, the WT hippocampus cells treated with PNU and Aβ; KN93+PNU+Aβ, the WT hippocampus cells treated with KN93, PNU and Aβ; KN93+PNU, the WT hippocampus cells treated with KN93 and PNU; KN93+Aβ, the control group treated with KN93 and Aβ. The Y-axis indicates the protein expression levels as a percentage of the control. The results demonstrated that the protein expression of CaMKIIα was significantly decreased in KN93 or Aβ oligomer-treated neurons, whereas expression of CaMKIIα was increased in the PNU groups (PNU+Aβ, KN93+PNU+Aβ and KN93+PNU). This result indicated that α7 nAChR activated the CaM-CaMKII-CREB signaling pathway. Data are presented as the mean ± standard deviation. ^*^P<0.05, ^**^P<0.01 vs. control; ^#^P<0.05, ^##^P<0.01 vs. Control+Aβ, ^△△^P<0.01 vs. Control+KN93.

To examine whether α7 nAChR activates the CaM-CaMKII-CREB signaling pathway, the present study measured the CaMKIIα protein level after adding KN93, which is a Ca^2+^/calmodulin-dependent protein kinase pathway inhibitor. As shown in Figure 12, compared with the control group, the levels of CaMKIIα were decreased in the Aβ and KN93 groups. Whereas, the expression level of CaMKIIα protein was significantly increased after PNU treatment. These results demonstrated that PNU282987 can activate the KN93-inhibited CaM-CaMKII-CREB signaling pathway. This result indicated that the neuroprotective effects of α7 nAChR is CaM-CaMKII-CREB signaling pathway dependent.

## DISCUSSION

AD is an intractable progressive neurodegenerative disease characterized by cognitive decline and dementia [20]. With the increase in the average life expectancy worldwide, the incidence of AD has also increased significantly [21]. Over the past few years, the potential therapeutic role of α7 nAChRs has been extensively studied [9, 22–24]. Using an agonist, such as PNU-282987, to modulate the activity of α7 nAChRs is a promising strategy to alleviate cognitive impairment [25]. However, the underlying mechanism of PNU-282987 on cognitive performance, synaptic change and its neuroprotective signaling pathway remains to be fully elucidated [26]. Thus, the aim of the present study was to evaluate the possible therapeutic role and underlying mechanism of PNU-282987 in synapse dysfunction and the neuropathological process of AD using both primary hippocampus cells and APP/PS1_DT mice models.

Following treatment with the a7 nAChR agonist PNU-282987, a specific α7 nAChR agonist with an efficacy of 100%, the deposition of Aβ was reduced, the brain weights were increased, and the learning and memory abilities were also improved in APP/PS1_DT mice both 6- and 10-months old. The expression levels of synaptic-associated proteins, including SYN, PSD95, SNAP25, DYN1 and AP180, were partially restored compare with the Aβ treated group. This result was in line with a study by Kroker et al, which found that an a7 nAChR agonist (SSR180711) was able to rescue impaired synaptic plasticity induced by Aβ infusion in hippocampal CA1 slices [27]. Additionally, transmission electron microscopy demonstrated that activation of a7 nAChR increased the number of synapses in hippocampal neurons of APP/PS1_DT mice, and the synaptic morphology was partially recovered, which could contribute to improving synaptic function and cognitive abilities. Immunohistochemistry and immunofluorescence experiments confirmed that the deposition of Aβ is negatively correlated with the expression of SYN and PSD95 proteins; therefore, the increase of SYN and PSD95 could decrease Aβ deposited in neural cells. Notably, the present study also found that the expression level of calcium signaling pathway proteins, including CaMKII, CREB, p-CaMKII and p-CREB, in PNU-282987 treated hippocampal neurons or the hippocampus of APP/PS1_DT mice was increased compared with in Aβ-treated groups. In summary, it has been demonstrated that activation of a7 nAChR by PNU-282987 could reduce the deposition of Aβ, alleviate its toxic effects on neural cells, reduce synaptic damage and activate the calcium signaling pathway. To the best of our knowledge, this is the first report of PNU-282987 decreasing the deposition of Aβ.

Several α7 nAChR agonists, such as GTS-21 [28], Encenicline [29] and Nelonicline [30], have also been considered as therapeutic drugs for AD. However, Encenicline use was halted in a clinic trial by the US Forum in 2016 due to its severe gastrointestinal response during the Phase III clinical phase. A peer-reviewed paper on the results of the monotherapy trial of Nelonicline revealed no significant improvement with any of the ABT-126 doses tested [28, 30]. As a novel agonist of nAChRs, GTS-21 stimulates the release of dopamine in the rat striatum and also enhances the learning ability of monkeys; however, it binds to human α4β2 nAChR 100 times stronger than that of α7 nAChR [28]. Compared with the aforementioned agonists, the main advantage of PNU282987 is that is binds to α7 nAChR specifically and activates α7 nAChR at an efficacy of 100%, thus it has been considered a promising candidate for improving the cognitive function of AD patients.

A pioneer study has shown that antagonists of a7 nAChR impair the performance of spatial memory in rodents [31]; therefore, numerous agonists of a7 nAChR have been tested for treating cognitive degeneration, and positive effects have been reported [24, 32, 33]. Nicotine activation of neurotransmitter release from presynaptic nAChRs elicits both long-term and short-term potentiating effects on synaptic plasticity [34] and enhances synaptic transmission [35]. Evidence has shown that 5 mg/kg PNU-282987 decreases motor activity in mice and appears to reverse stress effects [26, 36]. Another study has demonstrated that PNU-282987 can be used to improve cognitive symptoms in depression and schizophrenia [7]. The selective activation of a7 nAChRs by agonist has been reported to improve a variety of cognitive functions, including the attention of rodents and non-human primates, spatial learning, working memory and episodic memory [37, 38]. Additionally, activation of a7 nAChRs is considered to promote or prevent the damaging effects of Aβ on synaptic transmission [39]. Despite the ambiguity of the interaction of Aβ a7 nAChRs, preclinical evidence suggests that activation of a7 nAChRs restores behavioral defects associated with Aβ accumulation in animal models [9, 40]. In recent years it has been found that stimulating α7 nAChRs can promote not only neuronal excitation [41] but also attenuate Aβ-induced neuronal apoptosis [42]. Therefore, this improves spatial learning and memory abilities of AD patients [40]. The present study provides understanding of how a7 nAChRs exert a neuroprotective effect on an AD model, which may be by reducing the deposition of Aβ, restoring the expression of synaptic proteins and maintaining the synapse fitness, including the number and morphology.

**Figure 13:**
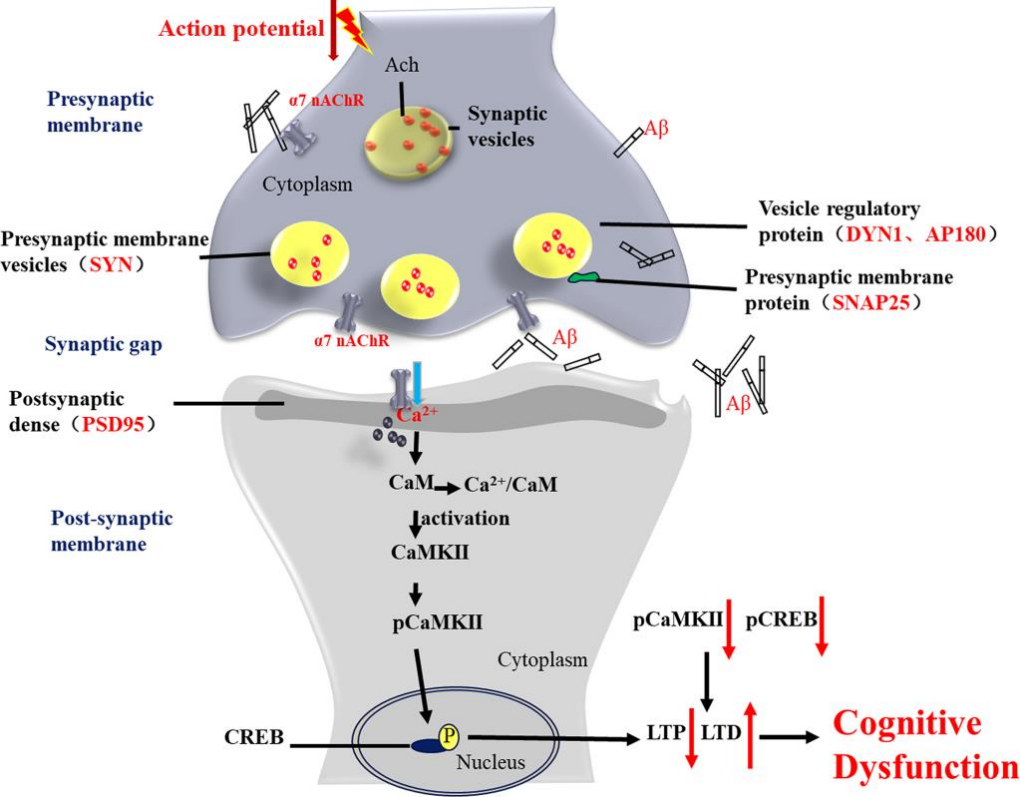
**Activation of α7 nAChR by PNU-282987 improves synaptic and cognitive functions through restoring the expression of synaptic-associated proteins and the CaM-CaMKII-CREB signaling pathway.** Synapses are the basic structure for the connection between neurons, and the function of a synapse is largely associated with synaptic-associated proteins. The present study found that activation of α7 nAChRs by PNU-282987 could largely restore the expression level of synaptic-associated proteins, including SYN, PSD95, SNAP25, DYN1 and AP180, which are downregulated by deposition of Aβ. α7 nAChRs belong to the ligand-gated ion channel coupled receptors, and form an ion channel in the center, which mainly regulates the flow of Ca^2+^ plasma inside and outside the cell. As a second cytoplasmic messenger, Ca^2+^ can flow into the cells through α7 nAChRs and bind to CaM to form an active CaM complex. The CaM complex can bind to CaMKII, which changes the CaMKII spatial conformation and phosphorylates CaMKII protein. Phosphorylated CaMKII enters the nucleus to catalyze CREB phosphorylation. CREB is located downstream of the α7 nAChR signal transduction pathway and acts as a transcription factor that regulates the growth and development of neurons and the formation of long-term memory.

Changes in Ca^2+^ homeostasis in AD patients are associated with synaptic dysfunction and neuronal decline. In a primary hippocampus cell model, the present study found that the expression levels of CaM-CaMKII-CREB signaling pathway-associated proteins were significantly reduced in neurons treated with Aβ. Activation of a7 nAChRs by PNU-282587 induced Ca^2+^ influx, which leads to intracellular Ca^2+^ overload and increases the expression of CaM. These results were consistent with a study by Haass et al [43]. In the hippocampus of APP/PS1_DT mice, the CaM-CaMKII-CREB signaling pathway proteins demonstrated a decreasing trend, and this trend became more significant as the age of the mice increased. A previous study has shown that activation of presynaptic α7 nAChRs by nicotine activates CaMKII, which triggers the long-term enhancement of glutamate release [44]. Activation of a7 nAChR by PNU-282987 increased Ca^2+^ influx and CaM expression level. As a result, the level of the Ca^2+^/CaM complex was increased. This may further activate CaMKII and the downstream CaM-CaMKII-CREB signaling pathway, thereby exerting neuroprotective effects on neural cells and significantly improving learning and memory abilities in APP/PS1_DT mice (Figure 13). In primary hippocampus neurons treated with the α7 nAChR antagonist MLA, certain CaM-CaMKII-CREB signaling pathway-associated protein expression levels were reduced, demonstrating to some extent the effect of PNU282987-specific agonistic α7 nAChR on this signaling pathway. However, changes in the expression of CaM protein were detected to be consistent with changes in agonistic α7 nAChR treatments, which may be related to cell processing *in vitro*.

The present study explored the possible therapeutic role and underlying mechanism of PNU-282987 in synapse dysfunction and the neuropathological process of AD, by using both primary hippocampus cells and APP/PS1_DT mice models. To the best of our knowledge, the present study provides comprehensive and in-depth understanding on the neuroprotective effect of α7 nAChRs and the underlying mechanisms. However, there are a number of limitations of the present study, such as the following: i) the animal and cell models may fail to fully mimic the pathogenesis of AD in human; ii) not all synapse-related and Ca^2+^ signaling-associated proteins were tested in the current study; iii) further studies should be designed to fully elucidate the neural protective effect of α7 nAChRs against the toxicity of Aβ; and iv) we didn`t monitor the status of the α7 nAChRs (activation or desensitization) in our experiment, however, the results suggested that low probability of desensitization and predominant activation may contribute the effect of PNU-282987 on α7 nAChRs.

In summary, the present study used an α7 nAChRs specific agonist PNU-282987 to active α7 nAChRs in both *in vitro* and *in vivo* models, and then analyzed its role in synapse morphology and functionality, the Ca^2+^ singling pathway and learning-memory abilities. The results indicated that activation of α7 nAChRs could reduce Aβ deposition in the hippocampus and protect neuron cells against Aβ toxicity. The protective mechanism of α7 nAChRs was proposed as follows: i) a decrease of the deposition of Aβ; ii) an increases expression of synaptic-associated proteins; and iii) maintenance of Ca^2+^ homeostasis by activation of the CaM-CaMKII-CREB signaling pathway. The present study may provide understanding on the underlying pathogenesis of AD.

## MATERIALS AND METHODS

### Reagents and antibodies

The following antibodies were used in the present study: Rabbit anti-SYN polyclonal antibody (cat. no. ab32594), rabbit anti-AP180 polyclonal antibody (cat. no. ab33898) and rabbit anti-p-CaMKIIα (Thr286) polyclonal antibody (cat. no. Ab5683) were purchased from Abcam (Cambridge, MA, USA). Rabbit anti-p-CREB (Ser133) monoclonal antibody (cat. no, 9198), horseradish peroxidase (HRP)-labeled anti-mouse secondary antibody (cat. no, 7076) and HRP-labeled anti-rabbit secondary antibody (cat. no. 7074) were purchased from Cell Signaling Technology, Inc. (Boston, MA, USA). Rabbit anti-PSD95 polyclonal antibody (cat. no. GTX133091), rabbit anti-SNAP25 polyclonal antibody (cat. no. GTX113839), rabbit anti-DYN1 polyclonal antibody (cat. no. GTX110379), rabbit anti-CREB monoclonal antibody (cat. no. GTX61140) and rabbit anti-β-actin polyclonal antibody (cat. no. GTX109639) were purchased from GeneTex, Inc. (Irvine, CA, USA). Mouse anti-CaM monoclonal antibody (cat. no. sc-137079) and mouse anti-CaMKIIα monoclonal antibody (cat. no, sc-13141) were purchased from Santa Cruz Biotechnology, Inc. (Dallas, TX, USA). GFAP (cat. no. ab7260) and NeuN3018822 were from Merck KGaA (Darmstadt, Germany).

Other reagents used in the present study were as follows: B-27 Additive, GlutaMAX Additive, NuPAGE LDS Sample Buffer (4X), NuPAGE Sample Reducing Agent (10X), NuPAGE Novex 4-12% Bis-Tris Precast, MES SDS Electrophoresis, Alexa Fluor^TM^488 goat anti-rabbit IgG antibody and Cy3^®^ goat anti-mouse IgG were obtained from Thermo Fisher Scientific, Inc. (Waltham, MA, USA). Hibernate-E Medium, DMEM high glucose medium and Neurobasal-A medium were purchased from Thermo Fisher Scientific, Inc. The ABC immunohistochemistry kit was purchased from Vectastain, USA. The primers for amplification of SYN, PSD-95, SNAP25, AP180, DYN1, CaM, CaMKII, CREB and β-actin mRNA were synthesized by Shanghai Genecore Bio Technologies, Shanghai, China. Aβ_1-42_, PNU-282987, HFIP (1,1,1,3,3,3-hexafluoro-2-propanol), DMSO and the other chemicals used were purchased from Sigma-Aldrich; Merck KGaA. MLA was purchased from Tocris Bioscience (Bristol, UK).

### Aβ oligomers preparation

Aβ was prepared according to a previously reported method [45]. Synthetic human Aβ_1–42_ was suspended in pre-cooled HFIP at a final concentration of 1 mM and then incubated at room temperature for 60 min, followed by incubation on ice for 10 min. Aliquots of Aβ solution were transferred into non-siliconized micro-centrifuge tubes, HFIP was allowed to evaporate overnight in a hood at room temperature, and the tubes were then stored at -80°C. Prior to treatment of the cells, the Aβ_1–42_ was dissolved in DMSO to obtain a final concentration of 5 mmol/l. For the preparation of oligomers, this solution was diluted with modified DMEM and then incubated for 24 h or at 4°C. Aβ oligomers were identified by western blot analysis.

### Primary neuron culture and treatment

Sprague-Dawley rats (within 24 hours of birth) were provided by the experimental animal center of Guizhou Medical University. The entire brain was immersed in pre-cooled D-Hank's balanced salt solution. The hippocampus was isolated, and the meninges and blood vessels were removed. Subsequently, it was cut into pieces with eye scissors and digested with 0.25% trypsin at 37°C for 15 min. The trypsin solution was then discarded and DMEM containing 10% FBS was added to terminate digestion. Following two washes with Hank’s buffered saline solution, the digestion was resuspended in 2 ml Neurobasal/B27 complete medium (Neurobasal A medium with 2% B27, 1% GlutaMAX supplement, 100 U/ml penicillin and 100 mg/ml streptomycin) and broken apart with Calibre Pasteur pipettes. Following removal of the supernatant, neurobasal medium supplemented with 2% B27 was added and gently pipetted 20 times. The supernatant cell suspension was gently pipetted, the cells were counted by trypan blue staining and cells were seeded on PLL-coated 6-well plates at a density of ~1x10 ^6^ cells/ml. The cells were then incubated in an incubator at 37°C with 5% CO_2_ and a saturated humidity, and half of the medium was replaced every 3 days. The immunofluorescence staining of glial fibrillary acidic protein (GFAP) and Hexaribonucleotide Binding Protein-3 (NeuN) was used to identify hippocampal neurons [46].

The primary neurons isolated were determined by double immunofluorescence staining with mouse anti-NeuN antibody (diluted 1:200), anti-mouse IgG labelled with CY-3 (red), rabbit anti-GFAP antibody (diluted 1:300) and anti-rabbit IgG labelled with FITC (green). After 10 days of incubation, the medium was replaced with neurobasal medium without the B27, and the neurons then were treated with Aβ oligomers (0.5 μmol/l) and/or PNU-282987 (10 μmol/l) and/or KN-93 (1 μmol/l) for 24 h. Cells were then harvested for subsequent experiments.

### Reproduction and identification of APP / PS1 double transgenic mice

Male and female APP/PS1_DT mice (B6. Cg-Tg) (weight, 20-30 g) were purchased from Shanghai Southern Model Biology Co., Ltd. (animal license no. SCXK 2014-0002). The genotypes of these mice were further confirmed by PCR to confirm the presence of the APPswe/PSldE9 gene mutations. Verification primers designed for APP and PS1 are listed in Supplementary Table 1. The expected lengths of the PCR products were 400 and 600 bp, respectively. APP/PS1 double transgenic mice were denoted APP/PS1_DT mice thereafter.

Different drugs/operations were applied to APP/PS1_DT mice to set up the following groups: i) WP group, wild-type C57 mice treated with PNU-282987; ii) AP group, APP/PS1_DT mice treated with PNU that were given daily intraperitoneal injections of 1 mg/kg PNU-282987 at the age of 6- and 10-months old for 5 days [26]; iii) APP/PS1 group, APP/PS1_DT mice injected intraperitoneally with the same amount of normal saline for 5 days; and iv) control group, wild type C57 mice injected intraperitoneally with the same amount of normal saline for 5 days. All operations on mice were approved by the Experimental Animal Ethics Committee of Guizhou Medical University (approval no. 1702153).

### Hippocampal tissue preparation

After opening the head skeleton and careful removal of adjacent, non-neural tissue, isolated hippocampal tissue was collected into a container and cooled on ice. Subsequently, 1 mm^3^ hippocampus was transferred into 4% glutaraldehyde solution for transmission electron microscope analysis. Brains were transferred into 4% paraformaldehyde in PBS (pH 7.4) for histological analysis, into ice-cold protein extraction buffer for western blot analysis or into TRIzol reagent for RT-qPCR analysis.

### Flow cytometry

Primary neuron cells were treated with Aβ, PNU-282987 (Aβ oligomers, 0.5 μmol/l; PNU282987, 10 μmol/l; KN93, 1 μmol/l) and MLA (100nmol/l) for 24 h. The cells were then washed three times with PBS and digested with trypsin for 3 mins. Digested cells were collected by centrifugation and suspended in binding buffer (BD Biosciences; FITC Annexin V Apoptosis Detection Kit I; cat. no. 556547) to a concentration of 1x10^6^ cells/ml. Annexin V-FITC dye and PI solutions were added to the cells and mixed gently, and then incubated for 15 min at 2-8°C in the dark. The treated cells were then immediately analyzed by flow cytometry.

### Morris water maze test

The Morris Water Maze test is widely used to study spatial learning and memory [47]. Mice in the different groups were subjected to four trials a day for total of 4 days, with a 5-7 min rest interval between trials. The movement of the mice was monitored with video track software (View Point, DNS-2). In each trial, the duration of a mouse to escape a platform was determined (escape latency). The mouse was allowed to sit on the platform for 5 sec after successfully locating it. Mice that failed to find the platform within 60 sec were manually guided to the platform, then allowed to settle on it for 20 sec, and the escape latency was recorded as 60 sec. The test was performed for 4 consecutive days. On the fifth day, the central platform was removed and the number of mice passing through the platform in 60 sec was recorded. Furthermore, the mice were subjected to a 60 sec probe trial, in which they were allowed to search for the location of the platform, and the time spent in each quadrant of the maze was recorded (quadrant search time). All these behavioral tests were conducted in a quiet environment with subdued lighting.

### Synapse morphology changes observed by transmission electron microscope

Brain tissue was fixed by 4% glutaraldehyde solution for >2 h and washed with 0.1 M PBS three times. Then samples were fixed by 1% osmium tetroxide for 2 h. Subsequently, the samples were dehydrated by gradient ethanol and acetone. Finally, the specimens were impregnated, embedded and polymerized by epoxy resin. Slices of 0.5 μm and 60 nm were prepared, and then doubled dyed with uranium acetate and lead citrate. A H-7500 electron microscope (HITACHI) was used to observe the changes of the synapse morphology in hippocampal neurons. The number of synapses, the width of the synaptic cleft, the thickness of synapse and the curvature of the synaptic interface were analyzed using an image analysis system (Image-pro plus 6.0).

### Analysis of the expression of synaptic-associated proteins and CaM-CaMKII-CREB signaling pathway proteins by RT-qPCR

The expression levels of synaptic-associated proteins (SYN, PSD95, SNAP25, DYN1 and AP180) and CaM-CaMKII-CREB signaling pathway proteins were determined by RT-qPCR, as described previously [48]. In brief, 1 μg total RNA extracted from the primary hippocampal cells was used as the template to reverse transcribe RNA to cDNA. RT-qPCR was performed in a 96-well with the ABI Step One Plus System and analyzed with SDS 1.4 (Applied Biosystems, Step One Plus Real-Time PCR system). These reactions were carried out with the universal TaqMan 2xPCR SYBR green mastermix in a 25-μl volume, which contained primers for analyzing the expression of synaptic-associated proteins. The relative transcript levels were calculated by the 2^-ΔΔCq^ method [49].

### Analysis of the protein levels of synaptic-associated proteins and CaM-CaMKII-CREB signaling pathway proteins by western blot analysis

The levels of the synaptic-associated proteins (SYN, PSD95, SNAP25, DYN1 and AP180) and CaM-CaMKII-CREB signaling proteins in tissues/cell lysates were quantified by western blot analysis as described previously [26]. In brief, total protein concentration was measured with the Coomassie brilliant blue protein assay. The proteins were separated by 10% SDS-PAGE and transferred to polyvinylidene difluoride (PVDF) membranes. Following blocking with 5% fat-free milk for 2 h, the membranes were incubated with primary antibodies against synaptic-associated proteins and CaM-CaMKII-CREB signaling proteins. The membranes were then washed and incubated with secondary antibody conjugated with HRP for 60 min. Finally, the proteins were visualized with the ECL chemiluminescence detection system (Syngene, GeneGnome XRQ NPC). Subsequently, the antibodies were washed off the membranes and the membranes were incubated with antibodies against β-actin (1:5,000) for 120 min and then with HRP-conjugated anti-mouse IgG for 60 min. The expression of the target proteins was calculated relative to the expression level of β-actin, which used as an internal control.

### Aβ1-42 and PSD95 expression analysis by microscopy and immunohistochemistry

Brain tissues were fixed by 4% paraformaldehyde in PBS for 24 h, In brief, 4% PFA-fixed and paraffin-embedded brain tissues were cut into 4-μm slices. The slices were then deparaffinized, rehydrated and incubated for 30 min in 3% H_2_O_2_. Subsequently, slices were unmasked using citrate buffer and then blocked with 5% normal goat serum for 30 min. Followed by incubation overnight with Aβ_1-42_ (1:100) and PSD95 (1:100) primary antibodies in a moist chamber at 4°C. For the detection of monoclonal antibodies, peroxidase-conjugated anti-mouse ABC kit (Vector, vectastain ABC KIT) was used. Negative control slides were incubated non-immune serum to replace the primary antibodies. The analysis was performed under a Nikon microscope with NIS-Elements AR software (Nikon, Model Eclipse Ci-E).

### Immunofluorescence detection of the relationship between the expression of SYN and Aβ1-42 in brain tissue

The right side of the brain was taken (including the hippocampus), fixed with 4% paraformaldehyde for >24 h, dehydrated, paraffin embedded and sectioned. The section was then dewaxed, citrate repaired, blocked with sheep serum working solution, and incubated with the corresponding primary antibody at 4°C overnight. After the primary antibody was discarded, the diluted secondary antibody (Cy3 labeled anti-mouse IgG and 488-labeled anti-rabbit IgG) was added dropwise, and the plate was sealed with DAPI after 1 h incubation at room temperature. The images were observed and collected using a fluorescence inverted microscope, and at least two fields of views were randomly selected for each group to be photographed 200 times. The background light of each photo was kept consistent. Image-Pro Plus 6.0 software was used to analyze the green and red fluorescence of each photo to obtain the integrated optical density (IOD) of each photo.

### Statistical analysis

All experiments were conducted three times and data are presented as the mean ± standard deviation (SD). The groups were compared using a two-tail Student’s t-test and one-way ANOVA using SPSS 22.0 software (IBM Corp., Armonk, NY, USA). P<0.05 was considered to indicate a statistically significant difference.

## Supplementary Material

Supplementary Materials

Supplementary Figures

Supplementary Table 1
