# Supplementary material for: Activation of α7 nAChR by PNU-282987 improves synaptic and cognitive functions through restoring the expression of synaptic-associated proteins and the CaM-CaMKII-CREB signaling pathway
Source: Aging (Albany NY). 2020 Jan 6;12(1):543–70. doi: 10.18632/aging.102640 (PMC6977648; doi:10.18632/aging.102640)
Supplement: Supplementary Materials [file aging-12-102640-s001..pdf]

SUPPLEMENTARY METHODS

Identification of APP/PS1_DT mice

To determine whether desired gene mutations occurred at APPswe/PSldE9 loci in the mouse model. Diagnostic PCR was used to identify the offspring of mice (primers were listed in Supplementary Table 1). Offspring produced both 400-bp and 600-bp amplicons were desired double-transgenic mutants for APP and PS1 genes. These mice denoted APP/PS1_DT were further used (Supplementary Figure 1). Previous research has found that the expression of α7 nAChR gene was not affected by APPswe/PSldE9 gene mutations in APP/PS1_DT mice [1].

Preparation of Aβ oligomers

Aβ protein has several different forms monomers, oligomers and fibrous bodies. It is widely recognized that soluble Aβ oligomers in the early stage of Aβ aggregation are the most neurotoxic, and play a key role in the pathogenesis of AD. To prepare Aβ oligomer from a commercially available monomer, we used synthetic human $A\beta_{1\text{–}42}$ in 1, 1, 1, 3, 3, 3- hexafluoro-2-propanol (HFIP). Western blot analysis showed that the prepared Aβ oligomers were mix of Aβ monomers (4KD, 18%), dimers (8KD, 2%), trimers (12 KD, 74%) and tetramers (16KD, 6%) (Supplementary Figure 2).

The purity of hippocampal neurons in primary bred rats

The in vitro isolated primary hippocampal neurons (NeuN) is an important model for studying the development and morphological function of neurons. We used immunofluorescence to identify the purity of hippocampal neurons (NeuN and GFAP antibody). The result showed that 81% of neurons cells were NeuN positive and GFAP positive (Supplementary Figure 3). This result indicated that the high purity in vitro neurons cell model was obtained.

REFERENCES

1. Inestrosa NC, Vargas JY, Arrazola MS, Rios JA, Carvajal FJ, Serrano FG and Farias GG. Nicotine Prevents Synaptic Impairment Induced by Amyloid-β Oligomers Through α7-Nicotinic Acetylcholine Receptor Activation. Neuromolecular Med. 2013; 15:549–69. https://doi.org/10.1007/s12017-013-8242-1 PMID:23842742
