# Supplementary material for: Activation of α7 nAChR by PNU-282987 improves synaptic and cognitive functions through restoring the expression of synaptic-associated proteins and the CaM-CaMKII-CREB signaling pathway
Source: Aging (Albany NY). 2020 Jan 6;12(1):543–70. doi: 10.18632/aging.102640 (PMC6977648; doi:10.18632/aging.102640)
Supplement: Supplementary Figures [file aging-12-102640-s003..pdf]

SUPPLEMENTARY FIGURES

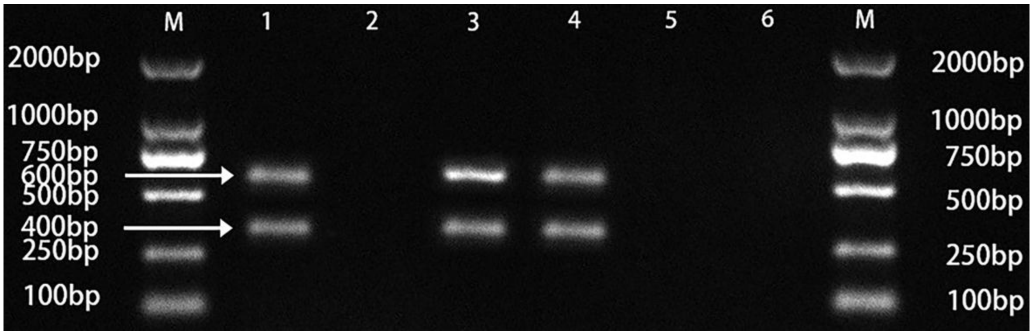

Supplementary Figure 1. Identification of APP/PS1_DT mice. Genomic DNA of all offspring mice were extracted from its tail and were used as PCR templates. Primers flank APP and PS1 loci were used to identify APP/PS1_DT mice form 1st generation bred offspring. Lane 1 – 6, PCRs using different genomic DNA as template, double-transgenic mice produce two distinct bands of 400 bp and 600 bp, while Control mice produces no band. Lane 1, 3, 4 were APP/PS1_DT mice, and lane 2, 5, and 6 were wild-type mice (note that only APP/PS1_DT mice will be subjected to further experiments). Lane M, DL2000 DNA marker (Takara, China).

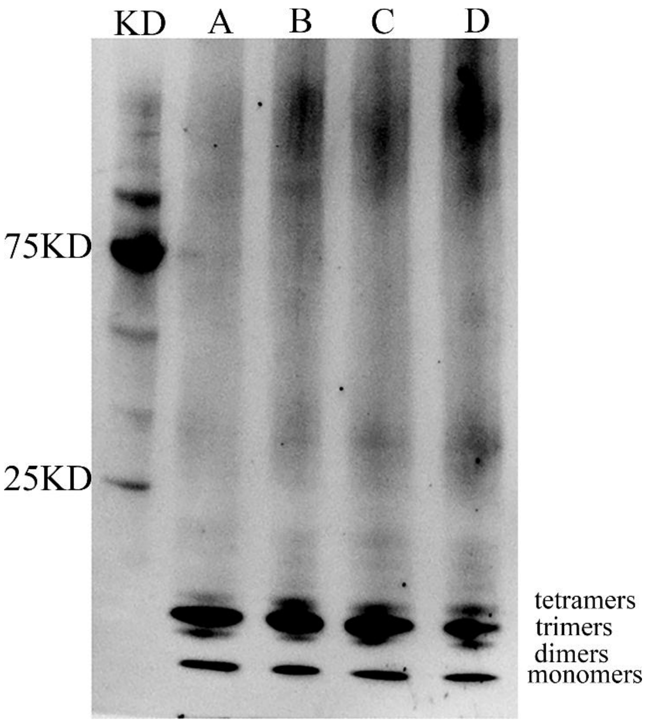

Supplementary Figure 2. Identify Aβ oligomers by western blot analysis. The first lane is protein weight marker, and lanes **A**, **B**, **C**, and **D** were Aβ samples. The monomer, dimer, trimer, and tetramer of Aβ were indicated at the right bottom. The results showed that we successfully produce a mixture of Aβ monomer (4KD, 18%), dimers (8KD, 2%), trimers (12 KD, 74%), tetramers (16KD, 6%).

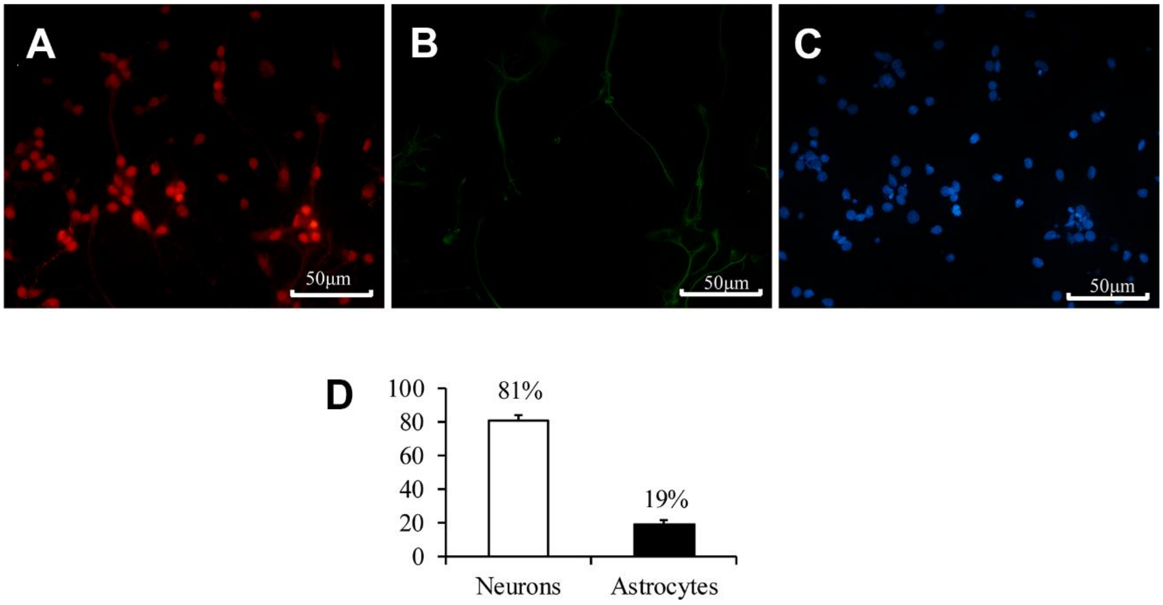

Supplementary Figure 3. Purity of primary hippocampal neurons in the APP/PS1 mice. Neurons (**A**, red); astrocytes (**B**, green); nucleus of neuron cells (**C**, blue) and the composition of neuron and astrocyte (**D**). As determined by immunofluorescent, the primary neurons isolated from 1st generation bred mice were approximately 81% of purity. Double staining of neurons and astrocytes were performed with mouse anti-NeuN and anti-mouse IgG labeled with CY-3 (neurons, red), and with rabbit anti-GFAP and anti-rabbit IgG labeled with FITC (astrocytes, green). The result indicated that the high purity of in vitro neurons cell model was obtained.

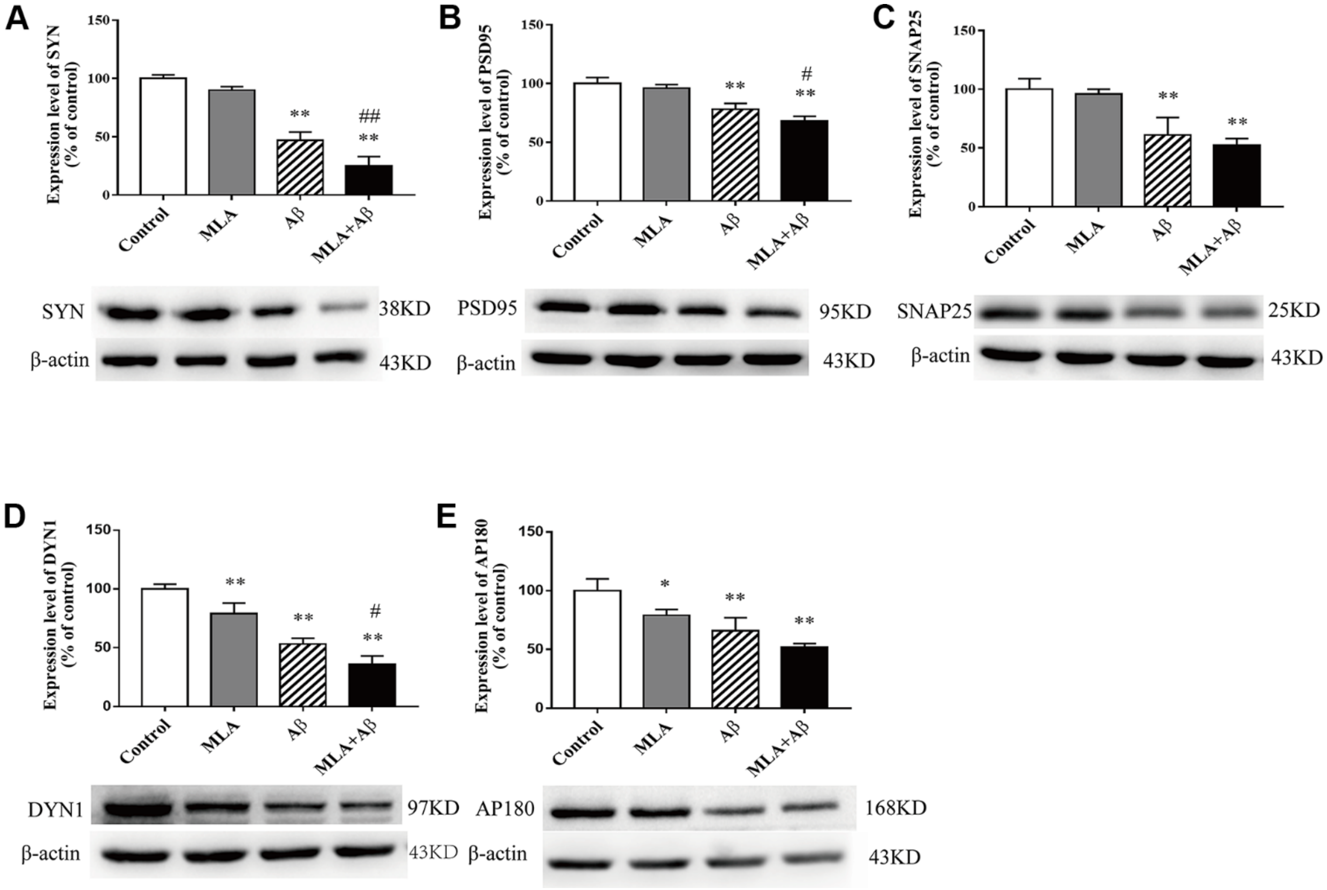

Supplementary Figure 4. Antiagitation of α7 nAChR decreases the expression of synaptic-associated proteins in Aβ-treated neurons. The X-axis labels are the neurons isolated from the WT rat (Control); the WT neuron cell treated with MLA (MLA); the WT neuron cell treated with Aβ (Aβ); and the WT neuron cell treated with MLA and Aβ (MLA+Aβ). The Y-axis indicates relative level of proteins (% of control). Detection of SYN protein (**A**); PSD95 protein (**B**); SNAP25 protein (**C**); DYN1 protein (**D**); AP180 protein (**E**); expression level in each group were measured western blot analysis (β-actin was used as an internal control). The results showed that the expression of SYN, PSD95, SNAP25, DYN1 and AP180 protein was significantly decrease in Aβ oligomers treated neurons, and this decreasing was enhanced by MLA treatment. Data are presented as the means ± SD. P<0.05, **P<0.01 vs. Control group; $^{\#}$P<0.05, $^{\#\#}$P<0.01 vs. Aβ.
