# Supplementary material for: Activation of α7 nAChR by PNU-282987 improves synaptic and cognitive functions through restoring the expression of synaptic-associated proteins and the CaM-CaMKII-CREB signaling pathway
Source: Aging (Albany NY). 2020 Jan 6;12(1):543–70. doi: 10.18632/aging.102640 (PMC6977648; doi:10.18632/aging.102640)
Supplement: Supplementary Table 1 [file aging-12-102640-s002..pdf]

SUPPLEMENTARY TABLE

Supplementary Table 1. Primers sequences used for identification of APP/PS1_DT mice.

Gene	Primer sequence (5′-3′)
APP	Upstream: GACTGACCACTCGACCAGGTTCTG
	Downstream: CTTGTAAGTTGGATTCTCATATCCG
PS1	Upstream: AATAGAGAACGGCAGGAGCA
	Downstream: GCCATGAGGGCACTAATCAT
